# Prof. Rau on the Mortality of Infants in the First Year of Existence

**Published:** 1839-04

**Authors:** 


MORTALITY OF INFANTS IN THE FIRST YEAR OF THEIR EXISTENCE. BY PROFESSOR RAU, OF BERN.

I. *Comparative Mortality of Infants.*

Of 2,808,139 deaths which took place in nine years, from 1820 to 1828, in the kingdom of Prussia, 751,077 were of children of one year old or less; that is to say, 26,944 in every 100,000. In Amsterdam, Paris, and in France generally, the proportion of early deaths is somewhat less formidable, as the following table will show:—Of 100,000 deaths,

| In France, in 1802. | In Paris, 1818-21, 1826-28, (6 years | In Amsterdam, 1816, 1818-29, (13 years.) | |
|---|---|---|---|
| 10,116 | 13,456 | 12,353 | were of children under three months, |
| 6,726 | 1,815 | 5,334 | ......... from three to six months, |
| 4,615 | 3,531 | 5,048 | ......... ... six to twelve ... |
| 21,457 | 18,802 | 22,735 | were of children of a year old or less.* |

* Bickes. Die Bewegung der Bevölkerung mehrerer Europ. Staaten. p. 69.

Of 100,000 deaths,

| In Sweden, in 1821-25, (5 years.) | In Westphalia and Rhenish Prussia, in 1820-28, (9 years.) | |
|---|---|---|
| 22,453 | 21,727 | were of children of a year old or less.* |

The mortality of infants is less in Paris than in France generally, on account of the number of foundlings who are removed into the country directly after their exposure at the Hôpital des Enfans trouvés: in the department of the Seine, in consequence of their thus being removed, the mortality is greater than in any other part of France.

From the above and other data, we learn that

| | | | |
|---|---|---|---|
| 26·69 | per cent. of the deaths in | Prussia | are of children under twelve months. |
| 21·72 | ...... | Rh. Prussia and Westphalia | ...... |
| 21·46 | ...... | France | .. ... |
| 29·45 | ...... | the departm. of the Seine | ...... |
| 22·45 | ...... | Sweden | ...... |
| 23·54 | .. ... | Courland | ...... |
| 18·80 | ...... | Paris | ...... |
| 22·74 | ...... | Amsterdam | ...... |
| 22·00 | ...... | Philadelphia | ...... |

According to Duvillard,† of 1,000,000 persons born in France, 767,528 only attain the age of a year. In the level provinces of Russia, 211 out of every 1000 die before the expiration of the first year; in Petersburg, 311; in Berlin, 276; in London, 320.‡

Of the whole number of children born,

| | | |
|---|---|---|
| 23·24 | per cent. in | Paris die before the expiration of the first year. |
| 20·02 | .. | Sweden. |
| 33·33 | ... | the province of Kasan (in Russia). |
| 21·10 | ... | Russia (in the level country). |
| 25·6 | ... | Berlin. |
| 32·00 | ... | London. |
| 31·1 | .. | St. Petersburg. |
| 16·65 | ... | Prussia. |
| 17·6 | ... | Courland. |

### II. *Comparative Mortality of the Sexes.*

Of the 751,077 children under a year old who died in Prussia in the nine years, 1820-28, 415,305 were boys and 335,792 girls.§ In Courland, the proportion of male to female infants dying under a year old is as 53·1 to 46·9;|| in Friesland, as 55·2 to 44·8;¶ in Paris, as 55·5 to 44·5.** The greater comparative mortality of male infants is in some measure only apparent, inasmuch as far more male than female children are born. It has been observed in Friesland, notwithstanding the disparity of male and female births, that at the age of forty the sexes were equal.††

### III. *Comparative Mortality of legitimate and illegitimate Infants.*

In Prussia, in the six years from 1826 to 1831, 17·56 per cent. of the legitimate children died before attaining the age of twelve months; and so many as 26·46 per cent. of the illegitimate.‡‡ According to Ramon de la Sagra, the rate of mortality of the legitimate and illegitimate children in the isle of Cuba is as follows: §§

* Bickes, p. 69.
† Friedländer, de l'Education physique de l'Homme. 1815, 8.
‡ Erdmann. § Bickes, p. 289.
|| Bidder. Beiträge zur medic. Statistik Kurlands.
¶ Coulon. Statistik Frieslands. **Frank, Med. Polizei, 795. Bd. v. s. 17.
†† Coulon. Op. cit. ‡‡ Bickes. Op. cit. Appendix, p. 41.
§§ Historia Economico-Politica de la Isla de Cuba, &c. 1831.

| | White Population. | | Coloured Population. | |
|---|---|---|---|---|
| | Legitimate. | Illegitimate. | Legitimate. | Illegitimate. |
| In the first week | 8·6 per c. | 7·1 per c. | 11·1 per c. | 14·3 per c. |
| From a week to a month | 2·6 ... | 6·5 ... | 4·4 ... | 5·0 ... |
| From one to two months | 1·7 ... | 2·8 .. | 2·5 ... | 2·2 ... |
| From two to three months | 2·7 ... | 4·1 ... | 2·1 ... | 2·2 .. |
| From three to twelve months | 11·4 ... | 7·5 ... | 13·9 ... | 11·7 ... |

### IV. *Influence of the Seasons on the Mortality of Infants.*

The season of the year evidently exercises great influence on the rate of the mortality of children. According to Trevisan, of 100 children born in Italy in the winter, 66 die in the first month, and only 19 survive the first year: on the other hand, of 100 born in summer, so many as 83 survive the first year; of 100 born in the spring, 48; of 100 born in the autumn, 58.* In Belgium, the rate of mortality in the first month, of children born in January, compared with that of children born in July, is as 33·21 to 17·19.† According to the researches of Villermé and Milne Edwards, published by Dumeril,‡ the mortality of children is much more considerable during the three winter months than during the rest of the year in France, in the southern parts of which country it diminishes in March, but in the northern not till April. In Philadelphia, however, the rate of mortality is very differently affected by the seasons: the season most fatal to children is from June to September, and the least fatal season is from November to January.§ In the island of Cuba, the rate of infant mortality is not sensibly affected by the change of the seasons.||

### V. *Proportion of Still-births to the total Number of Births in several European Countries.*¶

| | | |
|---|---|---|
| In Prussia, it is | 3·29 | per cent. |
| Sweden | 2·64 | ... |
| Saxony | 4·43 | ... |
| Hanover | 4·22 | ... |
| Mecklenburg Schwerin | 3·70 | ... |
| Sleswick and Holstein | 4·60 | ... |
| Petersburg (1803) | 0·20 | ... |
| (1806) | 0·70 | ... |
| Russia generally | 0·80 | ... |

There are more still-births of illegitimate than of legitimate children: it has been calculated that, where 3·166 per cent. of the legitimate births are still-births, 4·959 of the illegitimate are still-births.

According to the calculations of Bickes, of legitimate male children, 3·559 per cent. are still-born; of legitimate female, 2·749 per cent.: whilst, of illegitimate male children, 5·277 per cent. are still-born; and of illegitimate female, 4·632 per cent.** In Leipsic, during twenty-five years, from 1801 to 1825, the proportion of still-born to children born alive was as 1 to 17⅗; in 1822, in the district of Liegnitz, it was 1 to 15; in Arnsberg, 1 to 28; in Coblentz, 1 to 27; in Elberfeld, 1 to 17; in Magdeburg, 1 to 18; in Dusseldorf, 1 to 26; in Minden, 1 to 37; in Stralsund, 1 to 44½; in Erfurt, 1 to 30; in Merseburg, 1 to 21; in Posen, 1 to 49; in Berlin (in 1821), 1 to 19; in Gotha, 1 to 10; in Paris (in 1822), 1 to 20; in Saarlouis, 1 to 16; in Vienna, 1 to 36⅓. According to Casper, the average proportion of still-births to living births is as 1 to 19.††

*Ueber die unnatürliche Sterblichkeit der Kinder in ihrem ersten Lebensjahre.* Bern, 1836.

* Annali universali di Medicina, compilati da Omodei. Vol. xxxv. 1825.
† Quetelet et Smits. Recherches sur la Reproduction, &c. 1832.
‡ Bulletin des Sciences Méd. p. 188. 1829.
§ Emerson. Medical Statistics of Philadelphia.
|| Op. cit. Ramon de la Sagra.
¶ Bickes. Op. cit.
** Op. cit. p. 253.
†† Beiträge zur Med. Statistik, &c.

